# “Optimal” Central Venous Catheter Tip Position Does Not Increase Catheter Duration: A Retrospective Cohort Study

**DOI:** 10.7759/cureus.32627

**Published:** 2022-12-17

**Authors:** Rui Pereira, Francisco Seixas, Joana Almeida, Laura Gonçalves, Isabel Madeira, António Costa

**Affiliations:** 1 Anesthesiology and Critical Care, Centro Hospitalar Universitário do Porto, Porto, PRT; 2 Anesthesiology and Critical Care, Centro Hospitalar Universitário do Porto, porto, PRT

**Keywords:** catheter duration, catheter-related complications, central venous catheter (cvc), catheter-tip position, chest radiograph, thoracic ultrasonography

## Abstract

Background

Central venous cannulation provides venous access in different settings. Multiple guidelines and checklists still recommend confirmation of central venous catheter (CVC) tip position using a chest radiograph. The rationale is to detect and prevent complications thus optimizing CVC placement. Our primary hypothesis is that confirmation of catheter tip position by chest radiograph is not associated with increased catheter duration.

Methods

A retrospective cohort study was conducted with 921 patients included. Demographic, procedure and catheter data was obtained from adult patients that placed a CVC in the operating room. The catheter tip was independently classified as “optimal” or “malpositioned” independently by two researchers.

Results

Data from 921 CVC placements was collected. Patients who had a post-procedure chest radiograph (n=682, 74.0%) differed from those who did not in terms of co-morbidities (p=0.030), indication for CVC (p=0.023), duration of placement (p<0.001), number of punctured veins (p=0.036) and use of ultrasound (p<0.001). There was substantial agreement between researchers when classifying CVC tip as “optimal” or “malpositioned” (κ=0.632, p<0.001). No statistically significant difference was found between duration or complications of “optimal” CVCs compared to unknown tip/“malpositioned” CVCs. This study showed a 99% rate of clinically redundant chest radiographs according to Pikwer’s criteria for radiographic examination.

Conclusion

No difference was found regarding catheter duration or complications when comparing “optimal” and unknown/“malpositioned” tip. This study illustrates some consequences of post-procedure radiographs and reinforces that the risks/benefits should be weighed and that chest radiograph should not be done by routine.

## Introduction

Central venous cannulation is a procedure that provides venous access that can be used for the delivery of caustic medications and central venous pressure measurement. Moreover, central venous catheters (CVCs) are frequently required for patients needing parenteral nutrition, and often in patients where no other IV access is available. The procedure must be performed in strict aseptic conditions, can be aided by ultrasound, and may be required in a vast spectrum of medical or surgical patients. A chest radiograph (CXR) is routinely performed after the placement of a central venous catheter in many centers. The American Society of Anesthesiologists’ (ASA) 2020 guidelines recommend confirmation of the final position of the catheter tip using a CXR when the catheter is placed in the operating room [1]. Those same guidelines state that the correct catheter tip position can be confirmed with transthoracic ultrasound (same level of evidence) [1]. Other means of tip location include intracavitary ECG.

The main rationale defended for the CXR is to detect and/or prevent complications. Central venous catheter (CVC)-related complications can be classified as immediate or delayed and as mechanical, embolic, or infectious [2]. Those may arise from the cannulation technique or from an incorrect tip position, which is often impossible to unveil. Thus, the CXR is advocated to detect immediate complications such as pneumothorax and to identify an incorrect tip position which can in theory lead to complications.

The correct CVC tip position is a controversial topic. There is not a universally accepted correct position and different groups have diverse methods to identify a correctly placed CVC tip in the CXR [3-5]. Essentially, the debate is centered around the dangers of a “lower” CVC placement (right atrium) with the risk of cardiac tamponade and a “higher” CVC tip position associated with a higher risk of thrombosis [3].

CVC tip in the right atrium has been associated with an increased risk of cardiac tamponade [6]. There are, however, studies questioning this risk [7]. One study with 2348 intensive care unit (ICU) patients showed that patients with a CVC catheter tip in the right atrium did not have a greater risk of developing arrhythmias or cardiac tamponade when compared with patients with the tip in the superior vena cava (SVC) [8]. One prospective cohort study with 1619 patients concludes that the radiographic incidence of CVC malpositioning (extrathoracic or ventricular) is low and the clinical use of malpositioned catheters is associated with few complications [9]. Furthermore, there does not seem to be a correlation between SVC size and gender nor does the right superior heart border reliably identifies the atriocaval junction [10].

Pikwer et al. described a clinical decision rule based on the intended use of the CVC, aspiration of air during cannulation, and the presence of dyspnea/reduced oxygen saturation within eight hours to decide whether a patient would have a CXR control [11].

One retrospective study, in 1322 ultrasound-guided right internal jugular CVC attempts, at an academic tertiary care hospital system, over one year, showed a misplacement rate of 1.3% (95% CI, 0.8-2%) [12]. In 17 misplaced CVCs, four were left in place [12]. To detect pneumothorax, the authors also state that routine post-procedure CXR often resulted in false-positive results which led to additional unnecessary imaging [12]. Akin to this study, different authors also concluded that post-procedure CXR in uneventful CVC cannulations may be pointless [9,11]. Furthermore, several studies advocate the use of ultrasound, rather than CXR, to check for correct CVC tip position and exclude complications such as pneumothorax [13-15].

In our institution, if a temporary CVC is required for a patient in a level one ward, it is placed in the operating room (OR) by the anesthesia team. This procedure ensures safer working and aseptic conditions, but it also means that a whole team (nurses and anesthesiologists) and the physical space are occupied for that task. Ultrasound is readily available in the OR and is used in CVC cannulation at the discretion of the operator. On the other hand, to have a CXR in the OR a technician has to be previously requested. This study was developed, taking into account all the controversies regarding CVC tip position, to assess if the clinically relevant endpoint of catheter duration is changed by an “optimal” tip position. Some authors argue that correct tip position could lower catheter-associated complications, thus increasing its duration of use. Our primary hypothesis is that confirmation of short-term CVC tip position by routine CXR is not associated with an increased catheter duration.

## Materials and methods

In this retrospective cohort study, data was obtained from electronic files of a single-center academic tertiary hospital with around 850 beds. Adult patients that had a CVC placed in the OR, by request of the attending physician, from the 1st of January 2016 to the 31st of December 2019 were eligible. Exclusion criteria included patients that had (1) femoral CVC placement, (2) peripherally inserted central catheter (PICC), (3) totally implantable CVC, (4) CVC placed under fluoroscopic guidance, or (5) placed for anesthesia/ICU use. Data collected included demographic (age, gender, ASA score), procedure [CVC indication, duration (i.e., OR time), number of punctured veins, CVC site, ultrasound use, number of CXR, repositioning, CXR tip location], and catheter data (duration, removal reason, complications). The electronic files were reviewed until CVC removal, to record catheter duration and complications that may be associated with tip position/CVC cannulation.

Each catheter tip position was evaluated independently by two members of the research team. CVC tip was classified as “optimal” (upper, mid, and lower SVC and cavo-atrial junction), and “malpositioned” (jugular vein, brachiocephalic vein, right atrium, and other) based on a previous report [4]. If the tip position was not equally classified by the two members, a consensus was reached by the entire team. A CXR was classified as clinically redundant using a previously described clinical decision rule [11]. The study was approved by the Hospital’s Institutional Review Board (2020.142(112-DEFI/114-CE)).

Continuous variables were summarized with mean and standard deviation (SD) if they had a normal distribution or median and interquartile range (IQR) otherwise. Categorical variables were expressed with absolute frequency and percentage. Missing data were handled with pairwise deletion. Continuous variables were compared using Student’s t-test or Mann-Whitney U test, and categorical variables were compared using the Chi-square test or Fisher’s exact test as appropriate. Cohen's κ was applied to determine if there was an agreement between two independent observers regarding the tip position on the CXR. A p-value <0.05 was considered statistically significant. All statistical analyses were performed using SPSS Statistics 26.0 (IBM Corp., Armonk, NY, USA).

## Results

The retrospective analysis of data from the study period found 948 potentially eligible CVC placements. A total of 27 procedures were excluded from the analysis (12 femoral catheters, three PICC, and 12 fluoroscopy-guided catheters). Patient characteristics and indications for CVC are shown in Table 1. All patients were in a level one hospital ward at the time of CVC placement. The most common indication was the absence of IV access followed by the need for parenteral nutrition. Other indications included the use of chemotherapeutic agents or intensive treatment with other IV medications, dialysis, and the need for potassium supplementation. During the study period, 682 patients (74.0%) had a CXR after CVC placement. Data comparing the patients that had post-procedure CXR and those who did not are presented in Table 2. The two groups of patients differed on co-morbidities (p=0.030), indication for CVC (p=0.023), CVC site (p<0.001), duration of CVC placement (p<0.001), number of punctured veins (p=0.036), and use of ultrasound (p<0.001). No statistical difference was found regarding CVC duration or any complication in these groups. Other complications were nerve puncture and bleeding without hematoma. Missing data is also presented in Table 2. The total follow-up time was 13602 days with an average of 14.8 days per patient.

**Table 1:** Patient characteristics. SD – standard deviation; ASA – American Society of Anesthesiologists

Age, years, mean (SD)	61 (16)
Female gender, n (%)	501 (54.4)
ASA classification, n (%)	
I-II	127 (13.8)
III-V	794 (86.2)
Indication, n (%)	
Absence of IV access	498 (54.1)
Parenteral nutrition	284 (30.8)
Antibiotic therapy	89 (9.7)
Other	41 (4.5)
Vasoactive medication	9 (1.0)

**Table 2:** Comparison of patient, procedure and complication data by the performance of post-procedure CXR. SD – standard deviation; CXR – chest radiography; ASA – American Society of Anesthesiologists; CVC – central venous catheter Missing data, n (%): † CXR - 47 (6.9); No CXR - 33 (13.8) ͳ CXR - 53 (7.8); No CXR - 15 (6.3)

	CXR (n=682)	No CXR (n=239)	p
Age, years, mean (SD)	61.9 (16.1)	62.2 (15.8)	0.813
Female gender, n (%)	378 (55.4)	123 (51.5)	0.292
ASA classification, n (%)			0.030
I-II	104 (15.2)	23 (9.6)	
III-V	578 (84.8)	216 (90.4)	
Indication, n (%)			0.023
Absence of IV access	350 (51.3)	148 (61.9)	
Parenteral nutrition	227 (33.3)	57 (23.8)	
Antibiotic therapy	67 (9.8)	22 (9.2)	
Other	33 (4.8)	8 (3.3)	
Vasoactive medication	5 (0.7)	4 (1.7)	
Duration of procedure, minutes, mean (SD)	58.6 (25.8)	45.0 (17.4)	<0.001
Number of punctured veins, n (%)			0.036
1	654 (95.9)	236 (98.7)	
2+	28 (4.1)	3 (1.3)	
CVC site, n (%)			<0.001
Internal Jugular vein	360 (52.8)	215 (90.0)	
Subclavian vein	322 (47.2)	23 (9.6)	
Brachiocephalic trunk	0 (0.0)	1 (0.4)	
Ultrasound guided, n (%) ^†^	315 (49.6)	177 (85.9)	<0.001
Repositioned, n (%)	39 (5.7)	0 (0.0)	<0.001
CVC duration, days, median (min.-max.)	13 (0-84)	12 (1-103)	0.242
Reason for CVC removal, n (%) ^ͳ^			0.122
End of need/discharge	354 (56.3)	115 (51.3)	
Septic screening	119 (18.9)	39 (17.4)	
Infection	62 (9.9)	20 (8.9)	
Death	47 (7.5)	31 (13.8)	
Obstruction/malfunctioning	23 (3.7)	10 (4.5)	
Accidental removal	24 (3.8)	9 (4.0)	
Complications, n (%)			
Systemic infection	33 (4.8)	15 (6.3)	0.390
Local infection	11 (1.6)	3 (1.3)	1.000
Arterial puncture	19 (2.8)	3 (1.3)	0.182
Mechanical complication	16 (2.3)	5 (2.1)	0.821
Local pain	13 (1.9)	3 (1.3)	0.774
Hematoma	5 (0.7)	1 (0.4)	1.000
Pneumothorax	2 (0.3)	0 (0.0)	1.000
Other	3 (0.4)	0 (0.0)	0.572

Regarding CVC tip position, 95 CVC (14.0%) were classified as “malpositioned” after the first CXR. Following CXR, 39 patients (5.7%) had their CVC repositioned (including 22 classified by the authors as “optimal”), and only 13 repeated the CXR. Three patients (23.1%) from the group that had a second CXR still had a “malpositioned” CVC. At the end of the procedure, 83 patients had a “malpositioned” CVC and 26 had an uncertain location. There was moderate agreement between the observers regarding the anatomical location of the tip (κ=0.465, p<0.001) and substantial agreement when classifying the tip as “optimal” or “malpositioned” (κ=0.632, p<0.001).

There was no statistically significant difference (p=0.164) between the duration of “optimal” CVCs (median 13, IQR 13) and unknown tip/“malpositioned” CVCs (median 12, IQR 13). Even when compared with “malpositioned” CVCs (median 12, IQR 13) there was no significant difference (p=0.293). Regarding complications, no statistical difference was found when comparing “optimal” CVCs and “malpositioned” CVCs or with unknown tip locations.

Table 3 presents the catheter duration and complications detectable by CXR or those that may be related to CVC location/duration. Of the 682 patients that had a CXR, only seven met Pikwer’s criteria for radiographic examination hence there was a 99% rate of clinically redundant CXR. On the other hand, four patients had a CVC placed for vasoactive medication and did not have a radiographic control.

**Table 3:** Comparison of “optimal” and non-“optimal” CVC regarding catheter duration and complications. IQR – interquartile range; CVC – central venous catheter

	“Optimal” (n=573)	Unknown/“Malpositioned” (n=348)	p	“Malpositioned” (n=83)	p
CVC duration, days, median (IQR)	13 (13)	12 (13)	0.164	12 (13)	0.293
Complications, n (%)					
Systemic infection	29 (5.1)	19 (5.5)	0.792	2 (2.4)	0.287
Local infection	9 (1.6)	5 (1.4)	0.872	2 (2.4)	0.578
Mechanical complication	12 (2.1)	9 (2.6)	0.628	4 (4.8)	0.133
Pneumothorax	1 (0.2)	1 (0.3)	1.000	1 (1.2)	0.112

## Discussion

Central venous catheter placement is a universal procedure. It is often followed by a CXR that is advocated to locate the tip position and/or diagnose complications that could impair its use. The need for routine post-procedure CXR has been questioned for a long time [16-19]. Nonetheless, it still finds its place in guidelines for CVC placement [1,20,21].

Our study evaluated 921 catheter placements with a total of 13602 catheter-days. No difference was found regarding catheter duration or complications when comparing patients with “optimal” and unknown/ “malpositioned” CVC. Although there was no difference regarding complications, its low incidence limits the inferences that can be drawn.

**Figure 1:**
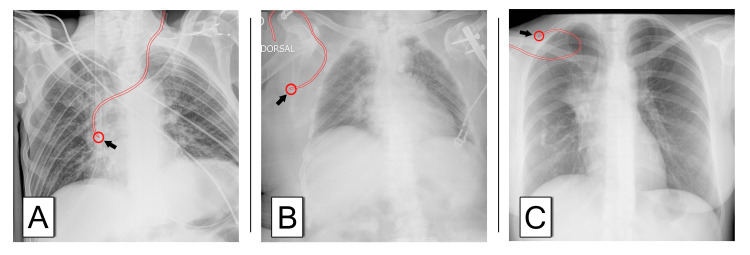
Post-procedure chest radiographs. Examples of post-procedure chest radiograph (CXR) recorded during the study period. (A) Indication: parenteral nutrition, duration: 84 days, removal reason: missing; (B) Indication: absence of IV access, duration: 10 days, removal reason: death; (C) Indication: chemotherapy, duration: two days, removal reason: end of need.

There are several controversies regarding “optimal” CVC tip. First and foremost, the ideal CVC position is still debatable [3-5,22]. Secondly, the association between CVC position and complications is not unequivocal [7,8,23]. Furthermore, as demonstrated in this study, a single incidence CXR may not be the best method to correctly identify the tip position. The evaluation of a CXR is subject to important inter-observer variability. The authors only had a moderate agreement (κ=0.465) regarding the anatomical location of the tip. Even when the tip was classified as “optimal” or “malpositioned” the agreement was only substantial (κ=0.632). Additionally, 22 catheters classified, by the authors, as “optimal” were repositioned following the post-procedure CXR showing the interobserver variability when examining a CXR. This can be explained by the lack of definitive landmarks and the difficulties in CXR interpretation found in daily practice. Figure 1 illustrates uncommon CVC positions and/or challenges found during CXR evaluation. This study also showed that, on average, 13.6 minutes of additional OR time were needed when performing a CXR.

Our primary goal focused on CVC duration as it is an important clinical endpoint. To the authors’ knowledge, no previous study reported the duration of CVC in the clinical setting. Short-term CVC can have numerous indications that range from the use of vesicant medications to the need of an IV line after exhaustion of peripheral access. Although named short-term CVCs, there is no clear limit to the maximal duration of these devices. In our sample, the maximum duration was 103 consecutive days. However, no difference was found regarding CVC duration when it was placed in an “optimal” position. Another highlight of this study is that it shows the practice of a tertiary center where every procedure was included, regardless of practitioner experience, thus showing a real-life setting.

However, the authors have identified some limitations. Firstly, this study is inherently prone to misclassification bias, namely recording errors or misinterpretation of records. Secondly, as the CXR was done at the description of the practitioner, selection bias may also be present. Indeed, the presented data show that the choice to perform a CXR differed by ASA classification, indication, number of punctured veins, choice of CVC site, use of ultrasound guidance and need for repositioning. Furthermore, as previously noted, this retrospective study may not have the sample size needed to detect complications associated to catheter placement and the reality in our center is likely different from most settings.

Nonetheless, our study underlines important consequences of routine post-procedure CXR such as increased procedure duration, use of resources, and radiation exposure both to the patient and the team. Our data add to the continuing controversy regarding the use of routine CXR following CVC cannulation. Clinical flowcharts may be helpful in reducing routine CXR and alternatives to CXR have been used successfully in clinical practice [11,13,14,24,25].

## Conclusions

No difference was found regarding catheter duration or complications when comparing “optimal” and unknown/“malpositioned” tip. The authors suggest that post-procedure CXR should not be done by routine. That is, the decision of performing a CXR should weigh the risks/benefits, taking into consideration factors such as indication, technique (jugular vs subclavian line), procedural difficulties (multiple punctures, arterial puncture, etc.) and complications (failure to aspirate blood from all lumens, aspiration of air, etc.). Also, clinical signs and symptoms after placement and alternative methods should be considered.

## References

[1] (2020). Practice guidelines for central venous access 2020: an updated report by the American Society of Anesthesiologists Task Force on central venous access. Anesthesiology.

[2] Smith RN, Nolan JP (2013). Central venous catheters. BMJ.

[3] Fletcher SJ, Bodenham AR (2000). Safe placement of central venous catheters: where should the tip of the catheter lie?. Br J Anaesth.

[4] Hade AD, Beckmann LA, Basappa BK (2019). A checklist to improve the quality of central venous catheter tip positioning. Anaesthesia.

[5] Wright D, Williams D (2020). Central venous catheter tip position on chest radiographs. Anaesthesia.

[6] Collier PE, Blocker SH, Graff DM, Doyle P (1998). Cardiac tamponade from central venous catheters. Am J Surg.

[7] Pittiruti M, Lamperti M (2015). Late cardiac tamponade in adults secondary to tip position in the right atrium: an urban legend? A systematic review of the literature. J Cardiothorac Vasc Anesth.

[8] Torres-Millán J, Torres-López M, Benjumea-Serna M (2010). Location of the central venous catheter tip in the right atrium: description in 2348 critical patients [Article in Spanish]. Med Intensiva.

[9] Pikwer A, Bååth L, Davidson B, Perstoft I, Akeson J (2008). The incidence and risk of central venous catheter malpositioning: a prospective cohort study in 1619 patients. Anaesth Intensive Care.

[10] Aslamy Z, Dewald CL, Heffner JE (1998). MRI of central venous anatomy: implications for central venous catheter insertion. Chest.

[11] Pikwer A, Bååth L, Perstoft I, Davidson B, Akeson J (2009). Routine chest X-ray is not required after a low-risk central venous cannulation. Acta Anaesthesiol Scand.

[12] Hourmozdi JJ, Markin A, Johnson B, Fleming PR, Miller JB (2016). Routine chest radiography is not necessary after ultrasound-guided right internal jugular vein catheterization. Crit Care Med.

[13] Megahed M, Habib T, Abdelhady M, Zaki H, Ahmed I (2018). Validity of ultrasonography in detection of central venous catheter position and pneumothorax compared with portable chest radiography. Res Opin Anesth Intensive Care.

[14] Smit JM, Haaksma ME, Lim EH (2020). Ultrasound to detect central venous catheter placement associated complications: a multicenter diagnostic accuracy study. Anesthesiology.

[15] Smit JM, Raadsen R, Blans MJ, Petjak M, Van de Ven PM, Tuinman PR (2018). Bedside ultrasound to detect central venous catheter misplacement and associated iatrogenic complications: a systematic review and meta-analysis. Crit Care.

[16] Lucey B, Varghese JC, Haslam P, Lee MJ (1999). Routine chest radiographs after central line insertion: mandatory postprocedural evaluation or unnecessary waste of resources?. Cardiovasc Intervent Radiol.

[17] Abood GJ, Davis KA, Esposito TJ, Luchette FA, Gamelli RL (2007). Comparison of routine chest radiograph versus clinician judgment to determine adequate central line placement in critically ill patients. J Trauma.

[18] Lessnau KD (2005). Is chest radiography necessary after uncomplicated insertion of a triple-lumen catheter in the right internal jugular vein, using the anterior approach?. Chest.

[19] Parmar MS (2021). (F)utility of "routine" postprocedural chest radiograph after hemodialysis catheter (central venous catheter) insertion. J Vasc Access.

[20] (2006). Clinical practice guidelines for vascular access. Am J Kidney Dis.

[21] Frykholm P, Pikwer A, Hammarskjöld F (2014). Clinical guidelines on central venous catheterisation. Acta Anaesthesiol Scand.

[22] Vesely TM (2003). Central venous catheter tip position: a continuing controversy. J Vasc Interv Radiol.

[23] Rutherford JS, Merry AF, Occleshaw CJ (1994). Depth of central venous catheterization: an audit of practice in a cardiac surgical unit. Anaesth Intensive Care.

[24] Gebhard RE, Szmuk P, Pivalizza EG, Melnikov V, Vogt C, Warters RD (2007). The accuracy of electrocardiogram-controlled central line placement. Anesth Analg.

[25] Saul T, Doctor M, Kaban NL, Avitabile NC, Siadecki SD, Lewiss RE (2015). The ultrasound-only central venous catheter placement and confirmation procedure. J Ultrasound Med.

